# Supplementary material for: A method to build extended sequence context models of point mutations and indels
Source: Nat Commun. 2022 Dec 22;13:7884. doi: 10.1038/s41467-022-35596-5 (PMC9780256; doi:10.1038/s41467-022-35596-5)
Supplement: Supplementary file 1 — Supplementary Information [file 41467_2022_35596_MOESM1_ESM.pdf]

Supplementary information for:

”A method to build extended sequence context models of point mutations and indels”

Jörn Bethune[1,*], April Kleppe[1,*] and Søren Besenbacher[1,2,#]

[1] Department of Molecular Medicine, Aarhus University, Denmark

[2] Bioinformatics Research Centre, Aarhus University, Denmark

[*] Contributed equally

[#] Corresponding author (besenbacher@clin.au.dk)

Contents:

Supplementary Figure 1 2
Supplementary Figure 2 3
Supplementary Figure 3 4
Supplementary Figure 4 5
Supplementary Figure 5 6
Supplementary Figure 6 7
Supplementary Table 1.......... 8
Supplementary Table 2.......... 9
Supplementary Table 3.......... 10

Supplementary Figure 1

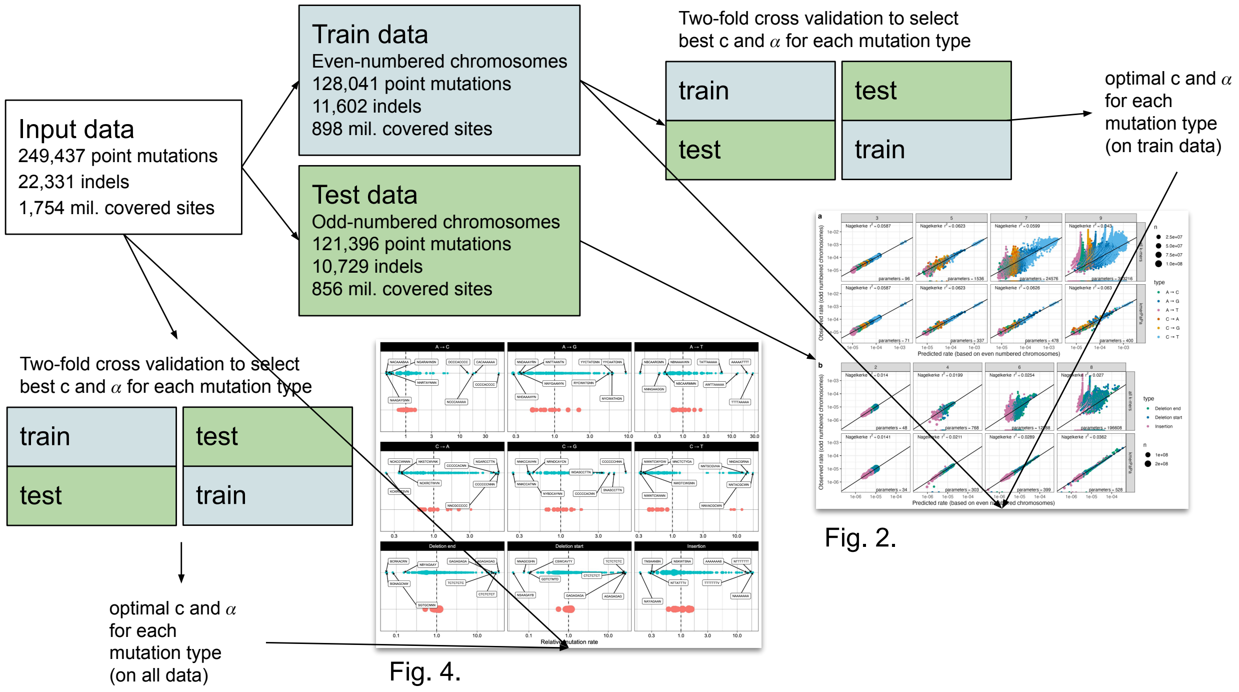

Supplementary Figure 1. Overview of the data used in different analyses.

Supplementary Figure 2

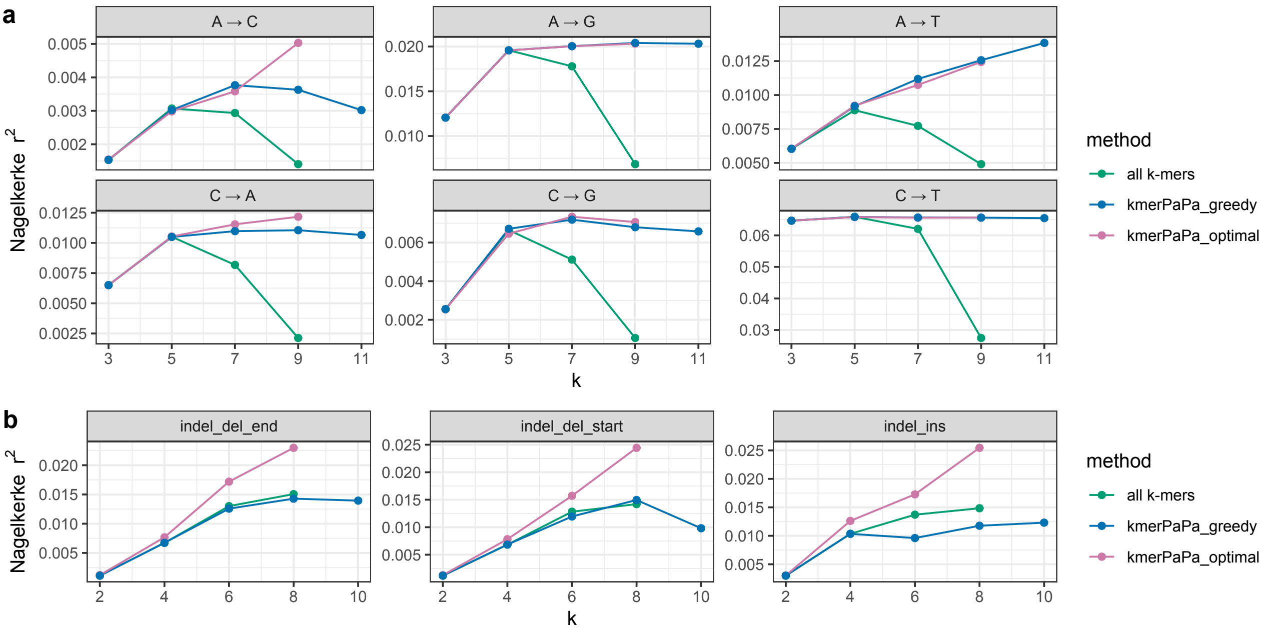

Supplementary Figure 2. Nagelkerke r^2 on test data for different values of k. Panel **a** show results for the 6 different point mutation types and panel **b** for the indel types. For long k-mers the "all k-mers" models overfit resulting in worse r^2 values on the test data. This is not the case for the kmerPaPa models. kmerPaPa-optimal is the algorithm used to generate the results presented in the article. kmerPaPa-greedy is a heuristic that balances the predictive performance with computational cost. The greedy algorithm is much faster for large values of k - but it also performs worse - especially in the case of indels.

Supplementary Figure 3

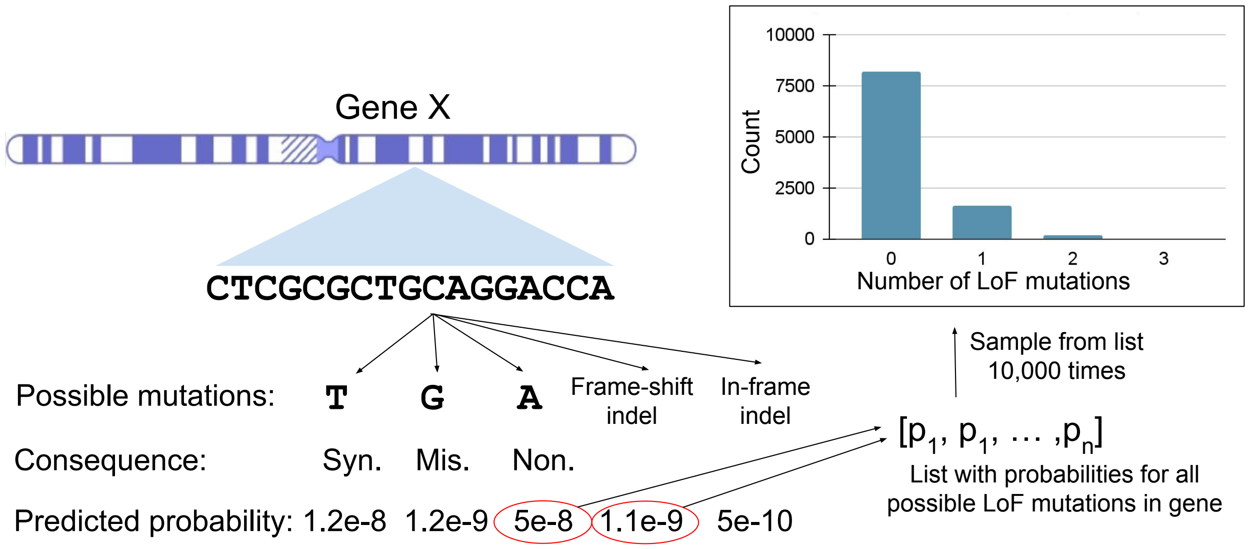

Supplementary Figure 3. Calculating the null distribution for the number of expected LoF mutations in a gene. Genovo considers all possible mutations in a gene and calculates their functional consequence. For a given mutation type (fx. LoF) it will then calculate a list of the probabilities for all possible mutations of this type. Genovo then samples from this list to create the null distribution that is used to calculate the p-values.

Supplementary Figure 4

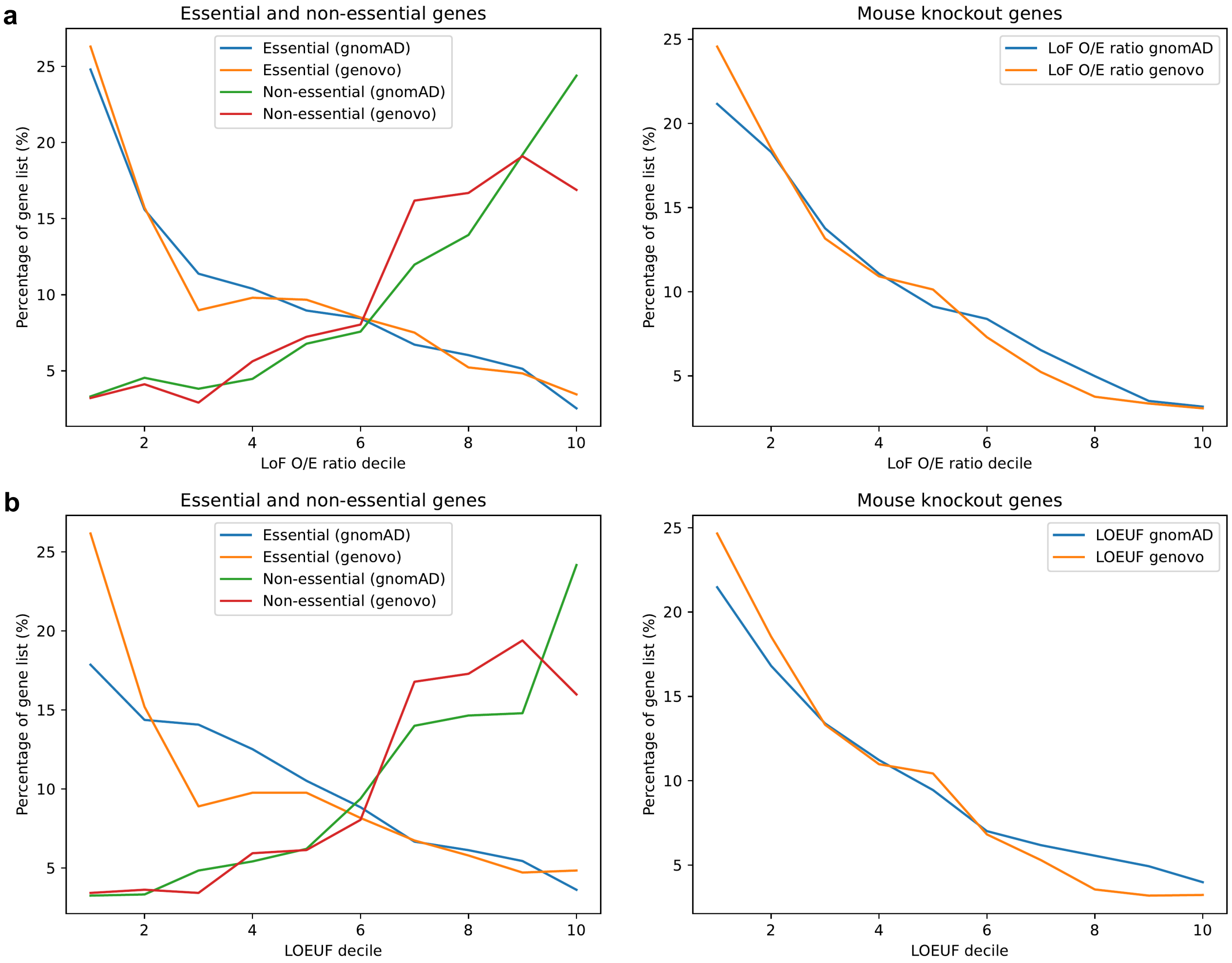

Supplementary Figure 4. Results from LOEUF analysis. In the first column we observe essential and non-essential genes. The green and blue lines depict the percentage of essential genes for Genovo and gnomAD, respectively. The red and yellow lines display distribution of non-essential genes for Genovo and gnomAD, respectively. The second column depicts essential knock-out genes for mice, and the third column depicts haploinsufficient genes. Panel **a** displays LoF observed/expected ratio, panel **b** depicts LOEUF score as inferred by gnomAD and Genovo.

Supplementary Figure 5

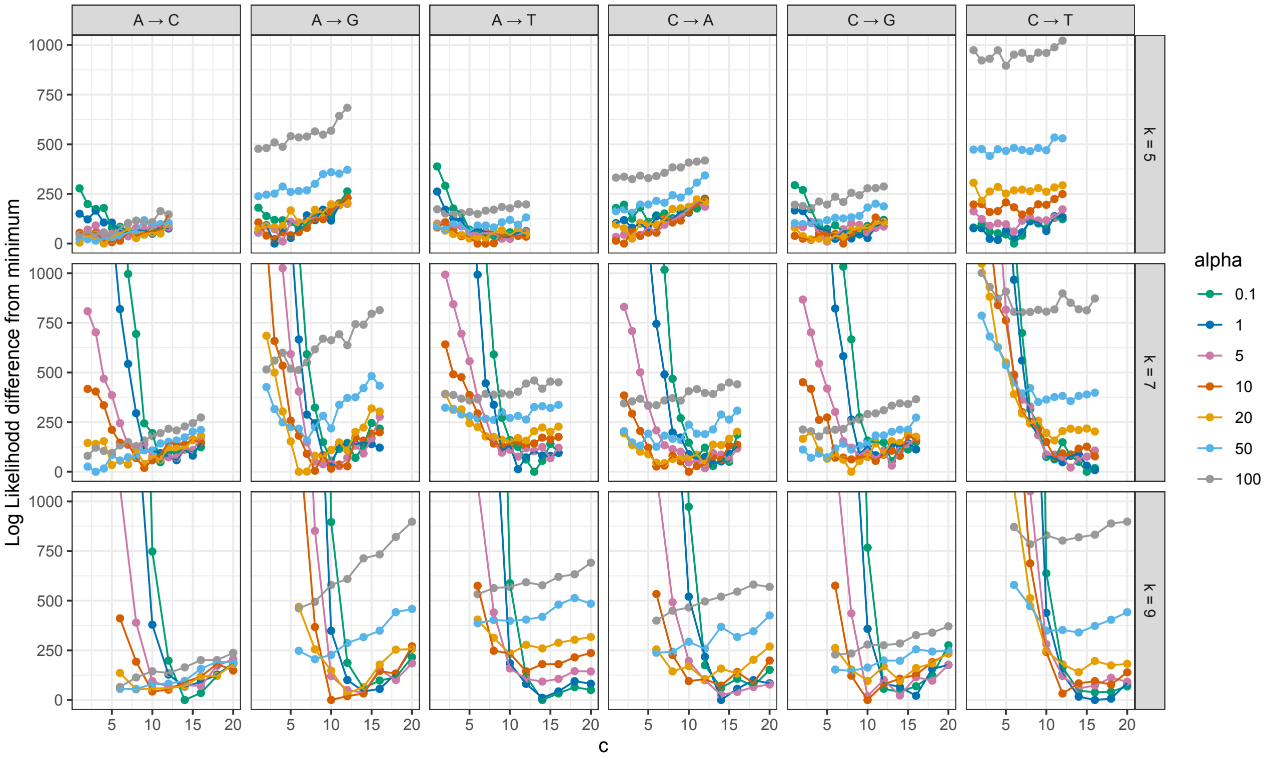

Supplementary Figure 5. Estimating hyperparameters. The plot shows the results of the grid search for the optimal combination of hyper parameters for each of the 5mer, 7mer and 9mer models. The y axis shows the Cross Validation Log-Likelihood for each parameter combination after subtracting the Log-Likelihood of the optimal parameter combination for each model.

Supplementary Figure 6

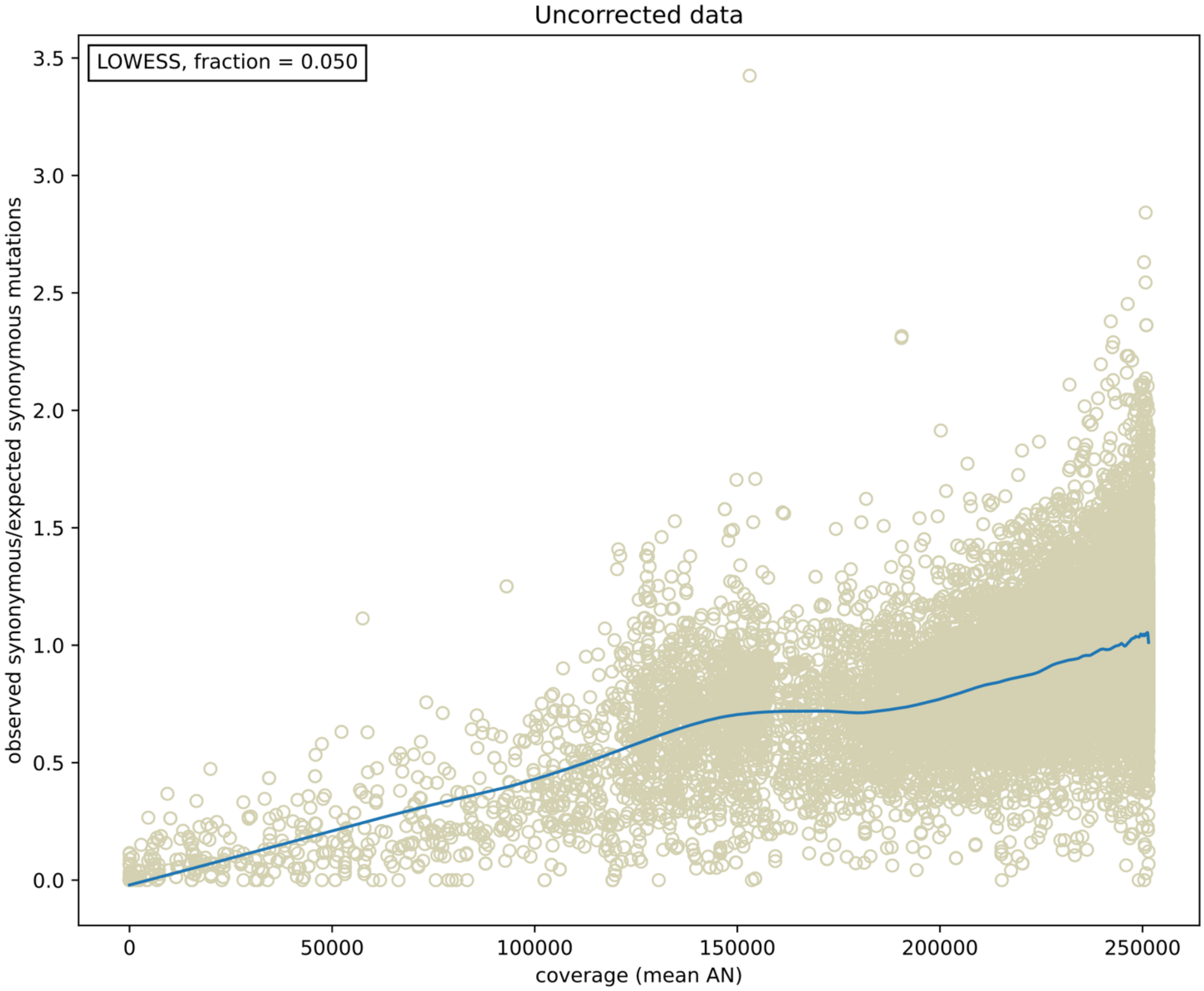

Supplementary Figure 6. Coverage Correction. Smoothed lowess values (blue line) based on the synonymous observed/expected ratio, over coverage for each gene (beige data). 'Fraction' is a number between 0 and 1, which represents the fraction of the data used when estimating each y-value.

Supplementary Table 1

Study	Trios	Autosomal point mutations	Autosomal indels	Reference Genome
Halldorsson et al. 2019 [1]	2977	194687	13367	hg38
Goldmann et al. 2016 [2]	816	35748	0	hg19
Francioli et al. 2015 [3]	258	11016	0	hg19
Sasani et al. 2019 [4]	471	26794	1892	hg19
Yuen et al. 2017 [5]	1652	104948	16582	hg19
Turner et al. 2017 [6]	1032	109433	3	hg19

Supplementary Table 1. Data sets with *de novo* mutations used as input to kmerPaPa.

Supplementary Table 2

Decile	% gnomAD	% Genovo
1	47.0	55.5
2	21.6	17.9
3	10.8	8.9
4	6.7	4.1
5	3.7	2.3
6	3.3	2.3
7	1.6	3.6
8	2.5	2.9
9	2.0	1.6
10	0.7	0.9

Supplementary Table 2. Percentage of haploinsufficient genes in each decile. Deciles clustered by Observed/Expected (O/E) ratio of Loss Of Function (LoF) mutations observed in haploinsufficient genes. Low numbered deciles (e.g. decile 1,2 or 3) contain the lowest O/E of LoF scores, whereas high numbered deciles contain higher O/E of LoF scores. These are the values plotted in Figure 6a.

Supplementary Table 3

Decile	% gnomAD	% Genovo
1	43.6	55.6
2	16.8	17.0
3	11.7	8.9
4	6.5	4.8
5	4.7	2.4
6	4.9	2.1
7	3.5	3.6
8	3.1	2.3
9	3.4	0.9
10	1.8	2.3

Supplementary Table 3. Percentage of haploinsufficient genes in each LOEUF decile. Low numbered deciles (e.g. decile 1,2,3 …) contain genes with the lowest LOEUF scores, whereas high numbered deciles contain higher LOEUF scores. These are the values plotted in Figure 6b.

Supplementary References

1. Halldorsson, B. V. *et al.* Characterizing mutagenic effects of recombination through a sequence-level genetic map. *Science* **363**, (2019).
2. Goldmann, J. M. *et al.* Parent-of-origin-specific signatures of de novo mutations. *Nat. Genet.* **48**, 935–939 (2016).
3. Francioli, L. C. *et al.* Genome-wide patterns and properties of de novo mutations in humans. *Nat. Genet.* **47**, 822–826 (2015).
4. Sasani, T. A. *et al.* Large, three-generation human families reveal post-zygotic mosaicism and variability in germline mutation accumulation. *Elife* **8**, (2019).
5. Yuen, R. K. C. *et al.* Whole genome sequencing resource identifies 18 new candidate genes for autism spectrum disorder. *Nat. Neurosci.* **20**, 602–611 (2017).
6. Turner, T. N. *et al.* Genomic Patterns of De Novo Mutation in Simplex Autism. *Cell* **171**, 710–722.e12 (2017).
