# Supplementary material for: A method to build extended sequence context models of point mutations and indels
Source: Nat Commun. 2022 Dec 22;13:7884. doi: 10.1038/s41467-022-35596-5 (PMC9780256; doi:10.1038/s41467-022-35596-5)
Supplement: Supplementary file 3 — Description of Additional Supplementary Files [file 41467_2022_35596_MOESM3_ESM.pdf]

File name: Supplementary Data 1

Description: kmerPaPa models trained on even-numbered chromosomes. These are the models depicted in Figure 2 and Supplementary Fig. 2.

File name: Supplementary Data 2

Description: kmerPaPa models trained on all chromosomes. These are the models used in Figure 3 and all the Genovo analyses.

File name: Supplementary Data 3

Description: The predicted and observed number of variants in GnomAD of each functional type for gencode v19 transcripts calculated using Genovo. The table also contains the observed and predicted number corrected to take the lower coverage of some transcripts into account and the calculated LoF O/E ratio and LOEUF score.
